# Cross-Shaped Heat Tensor Network for Morphometric Analysis Using Zebrafish Larvae Feature Keypoints

**DOI:** 10.3390/s25010132

**Published:** 2024-12-28

**Authors:** Xin Chai, Tan Sun, Zhaoxin Li, Yanqi Zhang, Qixin Sun, Ning Zhang, Jing Qiu, Xiujuan Chai

**Affiliations:** 1 Agricultural Information Institute, Chinese Academy of Agricultural Sciences, Beijing 100081, China; 2 Key Laboratory of Agricultural Big Data, Ministry of Agriculture and Rural Affairs, Beijing 100081, China; 3 Key Laboratory of Agro-Product Quality and Safety, Institute of Quality Standards & Testing Technology for Agro-Products, Chinese Academy of Agricultural Sciences, Beijing 100081, China

**Keywords:** zebrafish, digital phenotype, non-destructive examination, keypoints localization, deep feature learning

## Abstract

Deep learning-based morphometric analysis of zebrafish is widely utilized for non-destructively identifying abnormalities and diagnosing diseases. However, obtaining discriminative and continuous organ category decision boundaries poses a significant challenge by directly observing zebrafish larvae from the outside. To address this issue, this study simplifies the organ areas to polygons and focuses solely on the endpoint positioning. Specifically, we introduce a deep learning-based feature endpoint detection method for quantitatively determining zebrafish larvae’s essential phenotype and organ features. We propose the cross-shaped heat tensor network (CSHT-Net), a feature point detection framework consisting of a novel keypoint training method named cross-shaped heat tensor and a feature extractor called combinatorial convolutional block. Our model alleviates the problem of the heatmap-based method that restricts attention to local regions around key points while enhancing the model’s ability to learn continuous, strip-like features. Moreover, we compiled a dataset of 4389 bright-field micrographs of zebrafish larvae at 120 h post-fertilization for the model training and algorithm evaluation of zebrafish phenotypic traits. The proposed framework achieves an average precision (AP) of 83.2% and an average recall (AR) of 85.8%, outperforming multiple widely adopted keypoint detection techniques. This approach enables robust phenotype extraction and reliable morphometric analysis for zebrafish larvae, fostering efficient hazard identification for chemicals and medical products.

## 1. Introduction

Zebrafish (*Danio rerio*) are commonly employed in studying vertebrate gene function and diseases due to their high gene sequence similarity with humans [1,2]. Their larvae serve as an alternative model for screening drugs and chemicals for developmental toxicity [3]. The focus of research primarily lies on the organ regions of zebrafish [4,5]. However, with the rapid increase in the number of experiments using zebrafish larvae in recent years, there is an urgent need for an accurate, efficient, and high-throughput method to extract the organ features of zebrafish larvae.

Thanks to the nearly transparent characteristic of the epidermis of zebrafish larvae, their organ regions can be extracted from images through non-destructive means [6]. Prior phenotypic image analyses typically involved a single researcher visually assessing and scoring morphological features, which could be biased by the expertise and objectivity of the observer [6,7,8]. Deep learning techniques, especially convolutional neural networks (CNN), have significantly enhanced image processing performance and have found extensive use in plant and animal phenotype research [9,10], which is particularly relevant in biological research laboratories, where high-performance digital computing hardware is often lacking, specifically in regard to having no dedicated graphics processing unit (GPU) or high-performance central processing unit (CPU). In the published studies, CNNs were employed to analyze phenotype images in bright-field microscope zebrafish larvae images automatically [11,12,13]. However, these studies were limited to classifying particular phenotypes, necessitating using the same or similar instrumentation and settings as their established models. The studies’ universality and generalization ability were not ideal. Other studies attempted to extract general phenotypic features of zebrafish: identifying organ regions in zebrafish larvae automatically and then using the endpoint coordinates of organs for morphometric analysis and quantification [14,15]. CNNs allow the model to locate the feature points directly with clear semantic information. Therefore, skipping the organ region segmentation and directly locating the endpoint is feasible.

However, detecting feature endpoints in zebrafish larvae poses unique challenges that need to be addressed. The task of locating feature endpoints involves determining the position of specific features on the contour of zebrafish bodies and organs, similar to detecting keypoints in human pose estimation. Nonetheless, human pose estimation models are not designed to handle feature endpoints on the contour [16]. The human body’s keypoints encompass large regions of joints and organs, with highly structured semantic relationships but poor spatial feature robustness [17]. Consequently, these human body keypoint detection methods are less accurate for keypoints in small, narrow areas.

**Figure 1:**
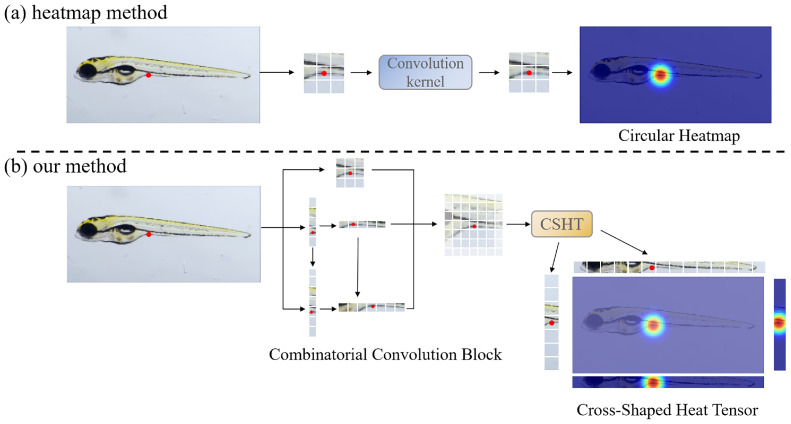
Comparison of the conventional keypoint detection framework and ours. (**a**) In the conventional framework for using a heatmap, the convolution kernel extracts the image feature map and obtains a local receptive field. Subsequently, the local field is compared to the heatmap point by point. The probability of keypoint occurrence is highest near the annotated position (red area) and gradually decreases as it moves farther away (blue area). (**b**) Our CSHT-Net, proposed in this study, breaks the strong connection between the model feature map and the GT label heatmap, allowing the feature map to explore long-distance information. Additionally, our network has augmented the receptive field using our improved combinatorial convolution block.

Although the human body keypoint method (as shown in Figure 1a) cannot be directly applied to the keypoints of zebrafish organ features, its structural framework still has a reference value for us when constructing the algorithm framework. A reliable keypoint detection framework usually involves three stages: data pre-processing, feature extraction, and keypoint detection, each playing a crucial role in the overall process [18]. The network’s performance in real-world scenarios is influenced by the alignment between the ground truth (GT) of keypoints and the output generated by the network. Heatmaps, widely used for GT matching in keypoint detection, transform each feature endpoint coordinate into a Gaussian probability distribution map [19]. In this map, the probability increases as values approach one [16], with σ representing the hyper-parameter that determines high-probability areas on the heatmap [17]. Studies like HigherHRNet [20] and SWAHR [21] suggested that each point should be paired with the optimal Gaussian kernel value to achieve the best model accuracy. The network structure is critical in keypoint detection, along with establishing a good heatmap. Researchers have explored various methods to improve keypoint detection accuracy, such as incorporating auxiliary tasks [22], dividing keypoints based on detection difficulty [23,24], and enhancing the capacity to learn high-resolution features or global information as well as the inter-relationships among various regions [25,26,27].

Our study meticulously develops an image-based convolutional keypoint detection framework that prioritizes zebrafish larvae morphometric endpoints. This approach ensures that keypoints appear along the edges and endpoints of organs, leading to high accuracy in identifying and measuring morphological characteristics in zebrafish larvae.

As illustrated in Figure 1, the proposed method differs from conventional human keypoint location techniques (as shown in Figure 1a). By employing the cross-shaped heat tensor, we mitigate the strong correlation between feature maps and heatmaps, allowing the network to represent feature point coordinates better and extract long-range semantic information (as shown in Figure 1b). To implement our cross-shaped heat tensor, we propose a network called CSHT-Net. We introduce a new feature extractor, the combinatorial convolutional block (CoConv-Block), which provides the network with multi-scale rectangular convolutional kernel combinations for extracting features that capture local and long-distance contour information. Our main contributions to this study are:

1. We propose a deep learning-based feature points location technique to represent zebrafish larvae morphology digitally. This approach focuses on describing the endpoints of organs with significant morphological features, thereby avoiding the impact of ambiguous and less important organ boundaries on the model.
2. We construct a dataset containing 4389 zebrafish larvae images in the lateral body position, wherein we accurately identify and annotate 26 distinct feature points.
3. We verify that the heatmap shapes and sizes influence the model’s positioning performance at edge feature points. The heatmap limits the keypoint detection model’s ability to extract global information.
4. We develop an advanced framework, CSHT-Net, which introduces a novel cross-shaped heat tensor method to replace heatmaps while enhancing the model’s capability to extract multi-scale rectangle information through our CoConv-Block. Comparative and ablation experiments on the constructed zebrafish dataset highlight the proposed CSHT-Net’s significant advantages.

This paper is organized as follows: Section 2 describes the acquisition process of the zebrafish dataset while providing a comprehensive explanation of the CSHT-Net. Section 3 presents the experimental results, and Section 4 discusses them. Finally, in Section 5, the paper concludes with a summary of the main findings.

## 2. Materials and Methods

### 2.1. Materials

#### 2.1.1. Zebrafish Larvae Image Acquisition

**Figure 2:**
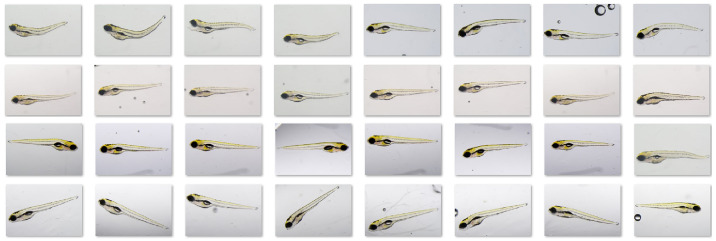
Examples of zebrafish larvae in our zebrafish dataset.

The existing zebrafish digital image datasets mainly focused on image classification or segmentation tasks and did not include datasets with keypoint annotations. We obtained the zebrafish larvae images used in the 26-keypoint study in a laboratory setting from samples with 120 h of embryonic growth. The zebrafish larvae samples were observed laterally, with their eyes overlapping in the direction perpendicular to the field of view and their bodies spread flat. Figure 2 displays some sample images from our dataset. The dataset contains 4389 images after cleaning. The training, validation, and test sets are divided into 8:1:1. Data annotation was done through the open-source labelme v5.4.1 software.

#### 2.1.2. Zebrafish Larvae Image Pose Normalization

**Figure 3:**
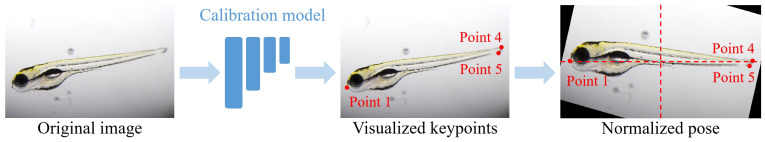
The process of zebrafish larvae image pose normalization.

The zebrafish larvae captured in the laboratory exhibit random poses and positions. This study introduces a pose normalization operation for zebrafish larvae to achieve uniformity in poses, sizes, and positions. Normalizing the original zebrafish images first can improve labeling accuracy. In Figure 3, the head and tail points (points 1 and 4) are transformed to locate on the horizontal center line of the image, with their midpoints aligned with the image’s center point, and the distance between them is adjusted to ensure equal length. For positional calibration, points 1 and 4 are utilized for rotation, translation, and scaling. Point 5, on the other hand, controls the flip of the zebrafish larvae in the image for morphological calibration.

The largest publicly available zebrafish image dataset we obtained [28] contains 1328 images with corresponding organ annotations. We selected 1019 zebrafish larvae images from this dataset and annotated points 1, 4, and 5 on these images for pose normalization. We used the high-resolution network with the highest-resolution channel number 32 (HRNet-W32) [25] to predict points 1, 4, and 5. The training and the validation datasets are divided into 8:2. The model takes RGB images as input and predicts the coordinates of these three feature points. The entire training process follows the standard HRNet human keypoint framework. The three-point detection model is less complex than a twenty-six-point model and requires less data, so we have easily obtained a three-point model with acceptable performance. The various models’ (HRNet-W32 [25] and residual network with 50 layers (ResNet-50) [29]) results are presented in Table 1.

#### 2.1.3. Feature Points Annotation of Zebrafish Larvae

**Figure 4:**
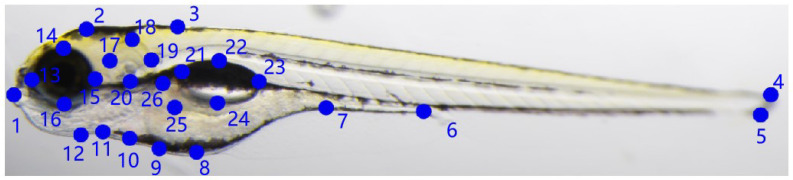
Visualized annotation example of zebrafish dataset with 26 feature points.

An image of zebrafish larvae with feature points is shown in Figure 4. The point coordinates are manually marked using the free and open-source program Labelme. The definition of feature points in this study is guided by three principles as follows: (1) Provide as much semantic detail as possible when describing the zebrafish larvae bodies and organs. (2) Reduce the number of feature points with similar semantic meanings. (3) Increase the number of feature points on parts with wavy contours. Table 2 provides a more detailed explanation of the definitions of zebrafish larvae feature points.

### 2.2. Methods

In this study, we replaced the conventional heatmap with a novel cross-shaped heat tensor (CSHT) method inspired by the soft label approach used in classification tasks. This method breaks the strong connection between the model’s feature map and the GT label heatmap, giving the network more flexibility to focus on long-range information. We introduced the CSHT-Net, incorporating the CSHT method for detecting zebrafish larvae endpoints. Specifically, the network’s feature extraction component utilizes an improved combinatorial convolutional block (CoConv-Block) to extract the contour features and filter noise.

#### 2.2.1. Cross-Shaped Heat Tensor to Represent Ground Truth

**Figure 5:**
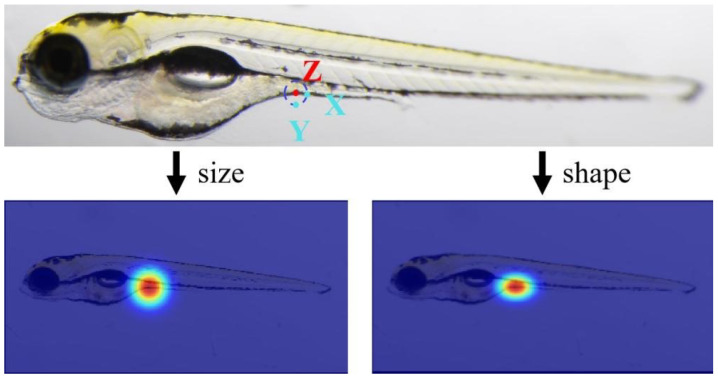
An example of keypoint prediction. Two predicted points, X and Y, for the GT point Z. The difference in human eye acceptance between two equidistant prediction points, X and Y, indicates that the heatmap’s size and shape influence the network’s performance.

The traditional heatmap is generated by applying 2D Gaussian kernels with constant variance $\sigma^{2}$ according to:

(1) $$\left\{\begin{array}{c} h\left(x,y,i\right)=e^{-\left(\frac{{\left(x-x_{i_{GT}}\right)}^{2}}{2\sigma^{2}}+\frac{{\left(y-y_{i_{GT}}\right)}^{2}}{2\sigma^{2}}\right)}\\ \left|x-x_{i_{GT}}\right|\leq 3\sigma \\ \left|y-y_{i_{GT}}\right|\leq 3\sigma \end{array}\right.$$

where $h\left(x,y\right)$ represents the probability value that the target point *i* appears at the coordinate $\left(x,y\right)$ in the heatmap, and $\left(x_{i_{GT}},y_{i_{GT}}\right)$ denotes the GT coordinate of feature point *i*. Different values of σ in various directions affect the network’s sensitivity to the feature point area. As illustrated in Figure 5, the predicted points X and Y are equidistant from the GT point Z. However, point X, parallel to the boundary (ZX) and closer to the contour, is more likely to be perceived as the true feature point by human eyes than point Y, which is perpendicular to the boundary (ZY). The discrepancy in the human eye’s level of acceptance between two equidistant prediction points, X and Y, suggests that the heatmap’s size and shape significantly influence the network’s performance. We will further validate this observation in the experimental section. Specifically, we designed an elliptical Gaussian kernel with unique parameter values in different directions, calculated as follows:

(2) $$\left\{\begin{array}{c} h\left(x,y,i\right)=e^{-\left(\frac{{\left(x-x_{i_{GT}}\right)}^{2}}{2\sigma_{x_{i}}^{2}}+\frac{{\left(y-y_{i_{GT}}\right)}^{2}}{2\sigma_{y_{i}}^{2}}\right)}\\ \left|x-x_{i_{GT}}\right|\leq 3\sigma_{x_{i}}\\ \left|y-y_{i_{GT}}\right|\leq 3\sigma_{y_{i}} \end{array}\right.$$

where $\sigma_{x_{i}}$ and $\sigma_{y_{i}}$ are the standard deviations of the elliptical Gaussian kernel for point *i*. We used a pair of unequal $\sigma =(\sigma_{x_{i}},\sigma_{y_{i}})$ with the σ in the parallel direction ($\sigma_{x_{i}}$) being greater than that in the vertical direction ($\sigma_{y_{i}}$). In this study, the long-side-σ value is set to one higher than the short-side-σ value. This anisotropic heatmap can reduce the influence of low-acceptance areas.

However, the heatmap method strongly associates the model’s attention with the output, thereby constraining the model’s perceptual capacity to the local region of keypoints. To address this issue, we propose a novel GT pre-processing technique called the cross-shaped heat tensor, which can be viewed as a length and width down-sampling of the heatmap.

**Figure 6:**
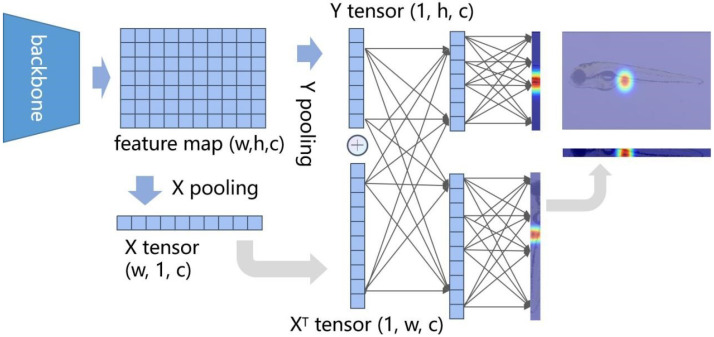
The structure of the cross-shaped heat tensor.

The heat tensor eliminates background noise while preserving important information along each axis. First, average pooling is applied to the feature maps to determine the height tensor and width tensor, as shown in Figure 6. Next, the vectors are transposed, unified in shape, and concatenated using a 1×1 convolution kernel to share feature information. After splitting the two vectors, each set of features is filtered. The width and height vectors can be converted back to a heatmap using simple vector multiplication. The method proposed is compatible with existing heatmap frameworks without excessive modifications.

#### 2.2.2. CSHT-Net

**Figure 7:**
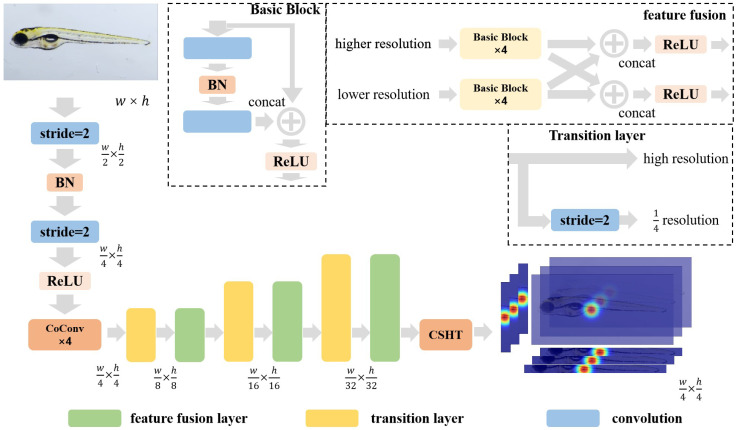
The architecture of our cross-shaped heat tensor network.

The architecture of the proposed CSHT-Net is demonstrated in Figure 7, with a detailed structural overview presented in Table 3. The network employs the 3×3 convolution kernel with a stride of 1 and uses rectified linear unit (ReLU) and batch normalization (BN) blocks to improve training stability. The variables *w* and *h* denote the width and height of the input image, respectively. The input zebrafish larvae image first undergoes two convolution kernels (Conv1 and Conv2) with a stride of 2, reducing the feature map size by 25%. The resulting feature maps are then processed through four CoConv-Blocks to extract relevant features. These output feature maps are subsequently passed through four alternating integration and transition layers incorporating features at various scales. Next, Conv3, with a 1×1 convolution kernel, reduces the output channel to 26, corresponding to the 26 zebrafish feature points. Finally, the 26 feature maps are down-sampled in the cross-shaped heat tensor into two cross-shaped heat tensors. BN and ReLU activation functions are applied at the end of these convolutional blocks to ensure training stability and introduce nonlinearity.

#### 2.2.3. The Structure of CoConv-Block

**Figure 8:**
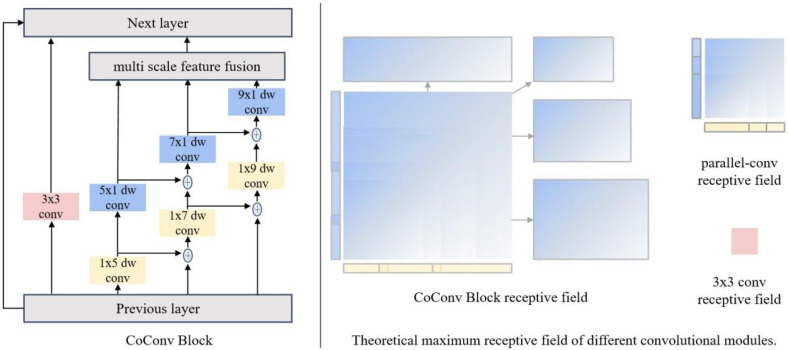
The structure of our CoConv-Block. It has a multi-scale branch, where a large branch can obtain input from both the previous layer and a small branch. Therefore, the receptive field of our CoConv-Block is much bigger than the original 3 × 3 convolution block and blocks that only parallel multi-scale convolution kernels.

As shown in Figure 8, our proposed CoConv-Block employs convolution kernels of various shapes, designed explicitly for elongated objects like zebrafish larvae, to capture context information. It connects parallel 1×5 and 5×1, 1×7 and 7×1, and 1×9 and 9×1 convolution kernels, which is particularly effective in extracting contextual contour features. To fuse multi-scale features, the output of the small-scale convolutional kernel is added to the input of the large-scale convolutional kernel. The traditional 3×3 kernel is also retained. Thus, our block can easily replace any block that uses a 3×3 convolution kernel. The output of all scale rectangular convolution kernels is superimposed with all feature maps using the following equation:

(3) $$output=f^{33}\left(x\right)+f^{51}\left(f^{15}\left(x\right)\right)+f^{71}\left(f^{17}\left(x+f^{15}\right)+f^{51}\right)+f^{91}\left(f^{19}\left(x+f^{15}+f^{17}\right)+f^{51}+f^{71}\right)$$

where *x* indicates $\frac{1}{4}$ of the previous layer’s output, $f^{xy}$ represents a convolution kernel of size x×y. For instance, $f^{51}\left(x\right)$ is the convolution kernel with a size of 1×5 and the input is *x*. The advantages of our CoConv-Block can be summarized as follows:

Prior knowledge prioritizes horizontal feature extraction. After standardizing zebrafish images, much information is concentrated horizontally. Prioritizing horizontal feature extraction can efficiently filter out valid information and remove background noise.

The strip-like receptive field captures rectangle information. Compared with the n×n kernel, the combination of 1×n and n×1 kernels prefers to learn linear features. If under the same parameter, the $1\times n^{2}$ kernel can learn information under a longer receptive field. Zebrafish have a strip-like body structure. In the early stage of feature acquisition, the strip-like receptive field can more efficiently capture body features.

Multiple strip-like feature chains. Internal shortcuts from small-scale kernels to large-scale kernels allow the combination of rectangular convolutional kernels of various shapes, widening the maximum receptive field and achieving rectangular receptive fields of different sizes.

As a result, the CoConv-Block effectively emphasizes crucial details, leading to a higher level of feature extraction by the convolutional layers.

#### 2.2.4. Feature Integration and Transition Layers

The alternately utilized transition and feature integration layers generate lower-resolution feature maps and integrate feature maps of different sizes. In CSHT-Net-L, the number of these two blocks and the sizes of feature maps are determined based on the HRNet-W48 settings. And in CSHT-Net-B, the settings follow HRNet-W32.

Finally, a 1×1 convolution reduces the channel of the final high-resolution feature map to 26, corresponding to the zebrafish larvaes’ feature endpoints. These feature maps are input into the cross-shaped heat tensor component for the final calculation.

#### 2.2.5. Evaluation Method

To evaluate the effectiveness of the networks, we adopted object keypoint similarity (OKS), which calculates the average distance between predicted points and ground truth points according to the following equation:

(4) $$\left\{\begin{array}{c} OKS=\frac{\sum_{i}e^{\left(\frac{d_{i}^{2}}{8s^{2}\sigma^{2}}\right)}\delta \left(\nu_{i}\right)}{\sum_{i}\delta \left(\nu_{i}\right)}\\ \delta \left(\nu_{i}\right)=\left\{\begin{array}{c} 1,\nu_{i}>0\\ 0,\nu_{i}=0 \end{array}\right. \end{array}\right.$$

where *d* represents the distance between the predicted point and ground truth point, *i* denotes the number of keypoints, σ represents the standard deviation of the keypoints, and $s^{2}$ is the scale factor of the image, which corresponds to the area of the target object in the image. Higher OKS values indicate better keypoint detection results. Since all 26 keypoints are visible in the zebrafish larvae dataset, the value of the $\delta \left(\nu_{i}\right)$ is constantly equal to one. To determine the formula for σ, we reverse Equation (5) to obtain Equation (6).

(5) $$\sigma =\frac{d}{2s\sqrt{-2lnOKS}}$$

where we set the OKS threshold equal to 0.75 (threshold value of AP.75). This threshold indicates that the tolerable error range of the keypoints in our work should be less than 1% of the maximum side length of the input image $\left(1\%\times max\left(w,h\right)\right)$. The image size $s^{2}$ is calculated using the zebrafish larvae bounding box $\left(max\left(x_{i_{GT}}\right)-min\left(x_{i_{GT}}\right)\right)\times \left(max\left(y_{i_{GT}}\right)-min\left(y_{i_{GT}}\right)\right)$. The value of σ is set to 0.0125.

We then used OKS to calculate the average precision (AP) and average recall (AR) to evaluate our network. In the context of zebrafish larvae feature endpoint detection, a positive prediction is defined as an OKS value higher than the threshold. All annotations are regarded as true annotations. An AP at 75% intersection over union (AP.75) represents the rate of the true-positive predictions among positive predictions when the error threshold is less than 1% of the input image $\left(1\%\times max\left(w,h\right)\right)$ in the test set. AP.5 corresponds to $\left(1.8\%\times max\left(w,h\right)\right)$, and AP.95 is $\left(0.5\%\times max\left(w,h\right)\right)$. mAP (or simplified AP) denotes the mean value from AP.5 to AP.95, which is more rigorous than AP.75. The AR is calculated similarly and represents the frequency of the true-positive predictions among true annotations.

#### 2.2.6. Experimental Settings

First, we normalized the zebrafish larvae pose in the images, and all images were resized to 384×288 pixels. Then, the models were trained to detect 26 zebrafish larvae feature points. The experiments were implemented using the PyTorch framework and were run on a Tesla A100 16G GPU. The batch size was 24 for all experiments, and the Adam optimizer was adopted. All models were trained for 120 epochs, with the learning rate set at 0.001, 0.0001, and 0.00001 at the beginning, 40th, and 80th epochs, respectively. Mean squared error (MSE) was used as the loss function between the predicted heatmap and the ground truth heatmap, which can be defined as:

(6) $$MSE\left(H,P\right)=\frac{\sum_{i}^{M}{\left(H_{i}-P_{i}\right)}^{2}}{M\times w\times h}$$

where *M* is the number of keypoints in the image, $H_{i}$ is the ground truth heatmap for keypoint *i*, and $P_{i}$ refers to the predicted heatmap for keypoint *i*. Note that $\left(H_{i}-P_{i}\right)$ is a pixel-wise calculation, and *w* and *h* represent the width and height of the heatmap, respectively.

## 3. Results

### 3.1. Using CSHT to Preprocess GT Is Superior to Heatmap

First, we investigated the impact of the shape and size of high-probability areas in the heatmap on the model’s performance. The size of these areas can be adjusted by fine-tuning the σ value. The average precision (AP) and average recall (AR) were employed to evaluate the performances of the networks. As shown in Table 4, when HRNet [25] is used as the base model with the isotropic heatmap pre-processing, a σ value of 3 achieves the best AP. This study shows that varying the heatmap size can result in differences of up to 0.4 AP and 0.7 AR. However, isotropic heatmaps fail to achieve significant shape modifications for high-probability areas. Table 4 also shows that an anisotropic heatmap generates differences of up to 1.3 AP and 1.4 AR compared to the isotropic heatmap.

To further verify the effects of the shape and size of the high-probability area, we expanded the values of σ in the model with σ being (4, 3) and exchanged the values of σ in both directions in the model with σ being (3, 2). Additionally, in the experiment with 50% σ being (2, 3) and 50% σ being 3, we decreased the number of points pre-processed with the anisotropic heatmap to half. The results show that changing the shape or reducing the number of points processed by the anisotropic heatmap generally leads to a performance decline. However, it still outperforms the best isotropic heatmap results. This study demonstrates that the shape and size of high-probability regions can influence model performance, indicating that a fine-tuned heatmap can significantly improve the model’s performance. These findings highlight the limitations of heatmap-based keypoint detection models regarding robustness and transferability.

To improve upon this, we propose the cross-shaped heat tensor to replace the original heatmap. The CSHT method achieves the best performance with 81.1 AP and 84.1 AR, increasing 2.3 AP and 2.0 AR compared to the base model. Unlike heatmaps, our CSHT module does not require fine-tuning the σ value, with the optimal value consistently being 1. The CSHT also shows better transferability and universality across different networks like the squeeze-and-excitation network (SENet) [26] and residual network (ResNet) [29]. As shown in Table 4, a CSHT with a default σ value of 1 outperforms the heatmap with the most appropriate σ value.

**Figure 9:**
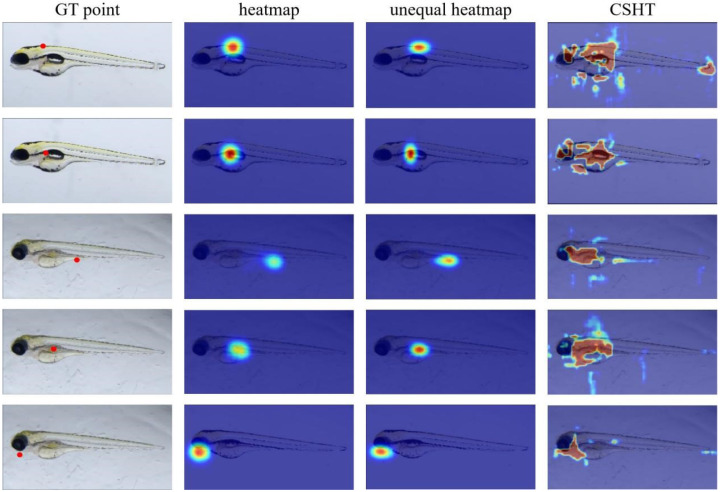
Visualized results of feature map generated by different pre-processing GT methods. Each row in the figure represents the output feature map of the same feature point. The first column displays the position of the ground truth of the feature point on the image. The second to fourth columns show the feature map from the model utilizing the heatmap, unequal heatmap, and our proposed CSHT technique. The attention in heatmap-based methods is confined to the area near keypoints, whereas with our method, the model’s attention expands to different parts of the zebrafish body. This proves that our method can free the model from the heatmap’s limitation on long-range feature extraction.

Additionally, we extracted feature maps using different preprocessing methods. Figure 9 depicts the high and low response values highlighted in red and blue, respectively. High response values indicate that the model emphasizes the region when localizing the feature endpoint, while common response values suggest the opposite. Feature maps extracted using different preprocessing methods reveal that the anisotropic heatmap generally produces lower overall response values and a smaller range of high response values compared to isotropic heatmaps. Isotropic heatmaps assume that feature points may appear in a larger area and are less confident about their appearance at each point, which suggests that isotropic heatmaps are not well-suited for zebrafish larvae. Especially in the fourth row from the top, the isotropic heatmap model produces a nearly elliptical feature map. This is similar to the classification model output, which does not strictly follow a one-hot tensor but has higher response values on similar categories. The heatmap predicted by the model gradually develops towards the heatmap based on the probability of semantic information. The probability value of the semantic information heatmap not only takes the distance from the GT as the basis but is also based on the similarity to the semantic information of the GT. From this perspective, the elliptical anisotropic heatmap created manually is better at aligning with the actual distribution of feature points in zebrafish larvae.

However, while the heatmap methods focus on the high-probability regions, they also limit the ability to utilize long-range information. In contrast, the CSHT can focus more on the central body region, which has semantic associations with almost all feature keypoints. The result indicates that our CSHT method successfully decouples the feature map and the keypoint heatmap. The semantic information is introduced into the keypoint decoder.

### 3.2. Comparison with Feature Extractor Blocks

In this section, we compare different blocks for network building. We follow the optimal bottleneck block number 4 in HRNet [25]. As shown in Table 5, the AP, AP.75, and AR are calculated, and the kilo parameter numbers (kParams) and Giga floating-point operations (GFLOPs) are shown to describe the model scale. The baseline model (a) is HRNet-W48 with the 3×3 convolution kernel, commonly applied due to its versatility. Given the elongated morphology of zebrafish, feature points are usually close to the body and organ boundaries. Thus, rectangular kernels are more appropriate for contour learning. The distinction in the following models lies in the structure of the block, which parallels the 3×3 kernel with various kernels. single-5 means adding the convolution 1×5 and 5×1 kernels string with the main branch 3×3 kernel to build the block. The results from the single- models indicate that broader kernels do not always improve the AP. The 1×5 and 5×1 convolution kernel string extracts the most contour features while minimizing the noise.

The Without- models incorporate multiple kernels, demonstrating the advantages of multi-scale rectangular convolution. Without-7 means adding without the convolution 1×7 and 7×1 kernels. The All-four model means adding all four branches into the block. However, additional context kernels do not always benefit feature point detection. Model Without-11 enhances performance more than the All-four model, indicating a preference for local context in feature point localization.

While more convolutional kernels theoretically improve feature extraction, excessive stacking is uneconomical. We propose an inner shortcut between similar convolutions, which achieves the effect of large kernels by combining small-sized convolutional kernels to achieve the effect of large kernels by combining smaller ones. Our network with the inner shortcut performs best when comparing the results of the Without-11 and CoConv-Block models. Because the internal shortcut needs to be implemented on multiple parallel convolutional kernels, there is no separate ablation experiment.

Our CoConv-Block is a plug-and-play module that can be connected in parallel to any network layer. In Table 6, we explore its contribution to network performance by integrating it into the bottleneck of HRNet. We folloed the optimal bottleneck block number 4 in HRNet [25]. Compared with the baseline model result, all models with improved blocks (squeeze-and-excitation (SE) block [26], convolutional block attention module (CBAM) block [30], coordinate attention (CA) block [31] and our CoConv-Block) show enhancement in the AP and AR. The CoConv-Block achieves the highest AP value of 81.4, outperforming other widely adopted blocks.

Furthermore, we adjusted the number of CoConv-Blocks. Table 6 shows that just one CoConv-Block achieves the optimal performance of other improved blocks with fewer parameters and computational requirements. Four CoConv-Blocks yield the model’s best AP.

### 3.3. Ablation Study on Different Components of CSHT-Net

An ablation study was conducted to assess the effectiveness of the cross-shaped heat tensor and the multi-scale combinatorial convolutional block within CSHT-Net. We removed these components separately and compared the results to the complete model. From the results of the ablation experiments presented in Table 7, the model incorporating both methods (model (d)) achieves the highest performance of 83.2 AP, 85.8 AR, and 95.5 AP.75. Conversely, models incorporating only one method (b and c) show a decrease in performance, indicating that each component contributes significantly to the final performance.

Overall, the ablation experiments provide insights into the relative importance of each component of the proposed method and highlight the effectiveness of the CSHT-Net for detecting the feature points of zebrafish larvae.

### 3.4. Comparison with Widely Adopted Methods

**Figure 10:**
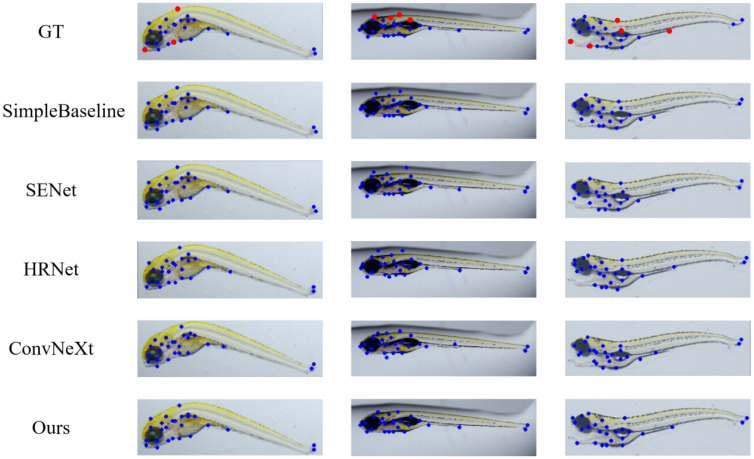
Visualized results of zebrafish larvae keypoint detection. From top to bottom, the corresponding feature point detection results are generated from ground truth, simpleBaseline, SENet, HRNet, ConvNeXt, and our network, respectively. The feature keypoints are marked with blue dots. The difficult-to-locate points typically found on the zebrafish larvae body contour are highlighted in red in GT.

We compared the proposed network with some mainstream keypoint detection methods, as summarized in Table 8 and visualized in Figure 10. SimpleBaseline [32] and HRNet [25] were selected as representative methods. SimpleBaseline incorporates de-convolutional layers [33] to maintain a higher-resolution feature map. As an encoder–decoder type framework, performance can be improved by advanced backbones. A deeper backbone yields better results for most vision tasks, with the AP increasing by 1.1% when transitioning from ResNet-50 to ResNet-152 [29]. The attention mechanism is another approach to achieving a comparable AP; using SENet-50 [26] as the backbone, the model achieves an AP similar to ResNet-152 while maintaining identical computing costs to ResNet-50. The SE-Block can extract features more efficiently than stacking network layers. ConvNeXt’s [34] poor performance in this task may be attributed to its large parameter size, which requires large amounts of data and multiple epochs of training, rendering it unsuitable for small datasets. HRNet-W48 achieves an impressive AP of 78.8% by preserving a high-resolution feature map during the feature extraction stages.

Our proposed CSHT-Net-L outperforms these methods, achieving the highest AP of 83.2% and AR of 85.8%. We have also developed a lightweight model, CSHT-Net-B, which achieves the second-highest AP while using half the computational resources of CSHT-Net-L. Given their lower number of parameters, lightweight models require a more remarkable ability to extract high-quality features. Compared to HRNet-W32 with similar parameters, CSHT-Net-B demonstrates a significant improvement of 10.0% in the AP and 9.0% in the AR, further affirming the effectiveness of our proposed method.

The visualization of three challenging-to-locate images and their respective feature points, predicted by various models, are presented in Figure 10. The hard-to-find points are usually situated along the contour of the zebrafish larvae body and are marked in red on the GT image. Zebrafish larvae with physical deformities are rarer in the dataset, so it is more difficult to locate key points, such as curved spines in the first column in Figure 10 and misshapen heads in the third. The second column is susceptible to interference from the background. The visualization results indicate that our model produces the most stable feature points within an acceptable range, even on morphologically abnormal zebrafish larvae. The CSHT method effectively decouples the strong correlation between feature maps and heatmaps, enhancing the network’s ability to extract long-distance semantic information and thereby improving feature point localization.

## 4. Discussion

In this study, we proposed CSHT-Net, a novel deep-learning framework designed for automatically localizing feature points in zebrafish larvae. Our approach addresses the limitations of traditional methods that focus solely on organ segmentation. We directly identified and analyzed organ endpoints with significant morphological features, providing a more accurate and reliable representation of zebrafish morphology.

Our approach is a simplified version of complete digitization, which is stable, practical, and easily applicable. Further research can investigate more aggressive feature point labeling quantities or organ segmentation using feature point connections between regions. Therefore, our work can be a fundamental basis for in-depth digital research on zebrafish larvae.

### 4.1. Exploring Universal Feature Representation for Zebrafish Larvae

Recent advances in CNNs have significantly improved automatic phenotype image analysis. However, challenges remain in enhancing model robustness and interpretability. Explaining the classification criteria remains unresolved, especially in end-to-end models with classification tasks.

Therefore, researchers in zebrafish quantitative studies used organ segmentation as a general feature representation method. CNN models handle feature extraction, while the classification criteria and interpretation are achieved through statistical methods or traditional machine learning models with better interpretability. However, the organ boundaries are not always evident when observing internal organs, leading to poor segmentation results. In practical applications, researchers select only a few distinct and easily recognizable edge points—usually the intersection points of two organs—across the entire organ segmentation region to construct the morphological features of zebrafish.

As a result, we propose skipping organ segmentation and directly using the filtered key feature points as the quantitative features for zebrafish analysis. Organ keypoints simplify organ region segmentation, focusing only on the endpoints of organs. This approach is more straightforward and offers strong robustness.

We developed CSHT-Net, a deep learning-based method specifically designed to locate feature points in zebrafish larvae and digitally represent their morphology. Our approach directly identifies the endpoints of organs with distinct morphological features. This bypasses the influence of indistinct or less critical organ boundaries, offering a more precise and relevant analysis.

### 4.2. Designing Edge Feature Point-Friendly Ground Truth Structures

Given the limited research on automatically identifying zebrafish larvae feature endpoints, we drew from advancements in human pose estimation and keypoint detection. However, human keypoint models are not ideally suited for zebrafish larvae datasets. In human models, keypoints are often centered within organs, allowing for some positional tolerance. However, feature endpoints are located at organ edges for zebrafish larvae. Therefore, more attention is required to local contour information and long-range semantic positional relationships.

Heatmaps treat all areas equally during training, which can lead to potential inaccuracies when focusing on smaller, localized semantic regions. Moreover, the heatmap area restricts the model’s receptive field output, limiting the model’s attention to the highlighted regions in the heatmap. As shown in Table 9 and Figure 1, the shape and size of the heatmap affect the model’s performance. Furthermore, we employed the CA module [31], a spatially aware attention module that can effectively adjust the model’s attention distribution. We replaced the baseline modules with the CA module at the input stage (where the CoConv block is located in Figure 7), at the intermediate feature fusion (feature fusion and transition layer in Figure 7), and at the output stage (where the heatmap and CSHT are used in Figure 7). As shown in Table 9, the performance improvement due to the attention module weakens as it grows closer to the output stage and even decreases performance at the heatmap stage. This further proves that the heatmap limits the diffusion of the model’s attention and affects its ability to capture long-range relational information.

Our CSHT method addresses this using vectorized ground truth heatmaps, enhancing the model’s focus on linear local semantic information. Inspired by label smoothing [35], we used a linear intermediate layer to isolate the model’s feature maps and decoder outputs. This allows for adjusting the output shape as needed while retaining the areas of interest for the model. Our two-dimensional vectors consider spatial dimensions, with elements representing the probability of keypoints at specific coordinates. This approach increases the density of local information and improves the model’s accuracy without needing hyper-parameter adjustments. The visual results confirm that our method effectively positions feature endpoints along organ edges.

### 4.3. Enhancing Multi-Scale Information Extraction

It is well known that convolutional networks learn more semantic information as depth increases, moving from local to global information. Self-attention also gradually expands from local to global regions with the transformer depth increases. Therefore, emphasizing local information near the model output will affect the overall acquisition of global information. However, the heatmap leads to the regions of interest in the output stage inevitably being confined to the vicinity of the keypoints. Therefore, keypoint detection models have to increase the receptive field near the input stage. At this stage, CNNs are more efficient than transformer structures [36]. On the other hand, high-performance digital computing hardware is often lacking in biological research laboratories. Transformers, which have higher hardware requirements (especially for GPU) than CNNs, were not considered.

The theoretical analysis of the module’s receptive field and the experimental findings demonstrate that using our suggested CoConv-Block has improved the model’s capacity to acquire global information on the input side.

### 4.4. Limitations of the Study

Despite achieving optimal performance, our study has limitations. First, actual usage scenarios require our model to operate offline on a computer without a discrete GPU, which restricts the models’ choice, scale, and performance. We hope our study can expand to more conducive usage scenarios for the model. Second, while our dataset comprises 4389 images, substantially larger than publicly available zebrafish datasets, it lacks multi-objective samples. Currently, our research is limited to single-target images. Our future work focuses on expanding the framework structure to a tensor-based key point network, which treats each point with a query tensor.

## 5. Conclusions

This study proposes a novel cross-shaped heat tensor network (CSHT-Net), a framework for automatically characterizing zebrafish larvae using well-designed feature key points. Our unified model has demonstrated superior performance compared to strong baseline models. This study can provide visual assistance for work focusing on the organ area of zebrafish larvae. How to extend our method to multi-target situations will be the focus of our follow-up research. This research has the potential to achieve efficient hazard identification of both chemicals and medical production using zebrafish larvae. Through in-depth research in our investigation, we hope to contribute to the nondestructive detection of phenotypic characteristics of zebrafish larvae organs and to detecting and studying biological and chemical products.

## Figures and Tables

**Table 1:** Comparison of the zebrafish larvae pose normalization model results. AP, AP.75, and AR are calculated. The parameter number (#Params) and Giga floating-point operations (GFLOPs) are also shown.

Model	Input Size	GFLOPs	kParams	AP	AP.75	AR
ResNet-50	256 × 192	8.99	33,996	0.985	0.990	0.990
**HRNet-W32**	256 × 192	**7.12**	**28,536**	**0.990**	**0.990**	**0.990**
HRNet-W32	384 × 288	32.89	63,595	0.990	0.990	0.990

**Bold** indicates that the model achieves the best performance on the metric.

**Table 2:** Detailed explanation of the 26 zebrafish larvae features points in the normalized pose.

Phenotypic Feature	Point Number	Semantic Information of Points
Body length	1	The front of the zebrafish’s muzzle
	2	The raised point on the head of the zebrafish
	3	The point of the first body segment of the zebrafish
	4	Position of the first vertex of the zebrafish caudal fin
	5	Position of the second apex of the zebrafish caudal fin
Yolk sac size	6	The end of the yolk sac extension
	7	The end of the area of the yolk sac projection
	8	The most convex point of the yolk sac
	9	The point at the junction of the pericardial sac and the position of the yolk sac
	25	The intersection of the pericardium and yolk sac
Heart size	10	The most concave or convex point of the lower edge of the pericardial sac
	11	The intersection point of the lower jaw and the pericardial sac
	26	The intersection point of the left lower edge of the bile site and pericardium
Lower jaw position	12	The most convex point of the lower jaw
Eye size	13–16	Points at the left, upper, right, and lower marginal positions of the eye
Ear size	17–20	Points at the left, upper, right, and lower marginal positions of the ear
Swim bladder size	21–24	Points at the left, upper, right, and lower marginal positions of the swim bladder

**Table 3:** The CSHT-Net architecture.

Layer Name	Kernel Size	Stride	Minimum Output Size	Minimum Output Channel	Repeat
Conv1	3×3	2	$\left(\frac{w}{2},\frac{h}{2}\right)$	64	1
Conv2	3×3	2	$\left(\frac{w}{4},\frac{h}{4}\right)$	64	1
CoConv-Block	multi	**1**	$\left(\frac{w}{4},\frac{h}{4}\right)$	256	4
integration layer	3×3	1	$\left(\frac{w}{32},\frac{h}{32}\right)$	48	4
transition layer	3×3	2	$\left(\frac{w}{32},\frac{h}{32}\right)$	48	4
Conv3	1×1	1	$\left(\frac{w}{4},\frac{h}{4}\right)$	26	1
CSHT	1×1	1	$\left(\frac{w}{2}+\frac{h}{2},1\right)$	26	1

**Table 4:** Comparison results for different GT pre-processing methods with different σ values to influence the network’s performance.

Model	Pre-Processing	σ	AP	AP.75	AR
HRNet-W48	Isotropic heatmap	1	78.4	93.3	81.4
		2	78.6	95.1	82.0
		**3**	**78.8**	**95.2**	**82.1**
		4	78.5	94.8	81.7
	Anisotropic heatmap	**2, 3**	**80.1**	**95.6**	**83.5**
		3, 4	**80.1**	95.4	82.9
		3, 2	78.8	95.5	82.1
		50% 2, 3 50% 3	78.8	95.3	82.5
	**Ours**	3	77.9	93.1	80.7
		2	79.1	94.4	81.8
		**1**	**81.1 (2.3↑)**	**95.3**	**84.1 (2.0↑)**
ResNet-50	Heatmap	2	78.0	93.9	81.8
	**Ours**	1	**78.8 (0.8↑)**	93.6	**82.1**
SENet-50	Heatmap	2	79.0	93.8	83.1
	**Ours**	1	**79.8 (0.8↑)**	**94.1**	83.0

**Bold** indicates that the model achieves the best performance on the metric. ^↑^ The values before the arrow represent the percentage improvement in the model’s performance evaluation metrics using our method compared to the model using the isotropic heatmap approach.

**Table 5:** Ablation study results for different kernel settings in our block. We followed the optimal bottleneck block number 4 in HRNet [25].

Model	1 × 5	1 × 7	1 × 9	1 × 11	Inner Shortcut	kParams	GFLOPs	AP	AP.75	AR
Baseline	×	×	×	×	-	63,596	32.89	78.8	95.2	82.1
**Single-5**	✓	×	×	×	-	**63,567**	**32.90**	**80.5**	**95.2**	**83.6**
Single-7	×	✓	×	×	-	63,568	32.91	79.2	94.1	82.2
Single-9	×	×	✓	×	-	63,568	32.91	79.4	94.2	82.2
Single-11	×	×	×	✓	-	63,570	32.92	79.3	94.5	82.2
Without-11	✓	✓	✓	×	×	**63,576**	**32.95**	80.9	95.3	83.7
Without-7	✓	×	✓	✓	×	63,578	32.96	80.7	94.6	83.1
All-four	✓	✓	✓	✓	×	63,582	32.99	81.0	95.3	83.7
**CoConv-Block**	✓	✓	✓	×	✓	63,610	**32.95**	**81.4**	**95.3**	**84.3**

**Bold** indicates that the model achieves the best performance on the metric.

**Table 6:** Comparison results with vital feature enhance blocks.

Block Name	Block Number	kParams	GFLOPs	AP	AP.75	AR
baseline model	4	63,596	32.89	78.8	95.2	82.1
+ SE block	4	63,976	32.88	79.9	94.1	82.8
+ CBAM block	4	64,005	32.88	80.4	93.7	83.1
+ CA block	4	63,603	32.96	80.6	95.0	84.0
+ **CoConv-Block (ours)**	4	63,610	32.95	**81.4**	95.3	84.3
+ CoConv-Block (ours)	1	63,388	31.56	80.6	95.2	**84.6**
+ CoConv-Block (ours)	3	63,535	32.49	81.1	95.3	84.4
+ CoConv-Block (ours)	5	63,681	32.42	81.2	**95.9**	**84.6**

**Bold** indicates that the model achieves the best performance on the metric.

**Table 7:** Ablation study on the design of our cross-shaped heat tensor network (CSHT-Net-L).

Model	CSHT	CoConv-Block	AP	AP.75	AR
(a)	×	×	78.8	95.2	82.1
(b)	✓	×	81.1	95.3	84.1
(c)	×	✓	81.4	95.3	84.3
(d)	✓	✓	**83.2**	**95.5**	**85.8**

**Bold** indicates that the model achieves the best performance on the metric.

**Table 8:** Comparison results for networks’ performance on the zebrafish dataset.

Model Name	σ	GFLOPs	kParams	AP	AP.75	AR
SimpleBaseline	2	21.85	34,002	78.0	93.9	81.8
SENet-50	**2**	20.25	36,533	79.0	93.8	83.1
ResNet-152	2	38.31	68,638	79.1	**95.7**	83.0
ConvNeXt-L	**2**	20.45	87,565	72.6	89.3	76.0
HRNet-W32	3	17.38	28,536	72.1	91.2	75.6
HRNet-W48	3	35.55	63,596	78.8	95.2	82.1
**CSHT-Net-B (ours)**	1	16.09	28,550	82.1	**95.7**	84.6
**CSHT-Net-L (ours)**	1	32.95	63,610	**83.2**	95.5	**85.8**

**Bold** indicates that the model achieves the best performance on the metric, while underlining indicates the second-best performance.

**Table 9:** Comparison results for various positions of the CA block.

Block Position	kParams	GFLOPs	AP	AR
Baseline	63,596	32.89	78.8	82.1
+Input	63,603	32.42	80.6	84.0
+Middle	64,002	32.90	80.4	83.7
+Output	64,015	32.98	74.3	76.6

## Data Availability

Our demo, training codes, and datasets are available on GitHub: https://github.com/starduct/CSHTNet (accessed on 26 December 2024).
